# The New York Institute of Stomatology

**Published:** 1898-07

**Authors:** 

**Affiliations:** The New York Institute of Stomatology


### Reports of Society Meetings.

### THE NEW YORK INSTITUTE OF STOMATOLOGY.

A REGULAR meeting of the Institute was held on the afternoon and evening of Tuesday, May 3, 1898, at the Windsor Hotel, Fifth Avenue and Forty-sixth Street, the President, Dr. E. A. Bogue, in the chair.

### AFTERNOON SESSION.

*The President.*—It is with great pleasure that I welcome the gentlemen who are our guests to the discussion of two subjects which are of importance to us all. The subject chosen for this session is "Journalism," while we will turn our attention this evening to the subject, "Secrets and Patents." I take pleasure in presenting Dr. Wm. H. Potter, of Boston, who will read a paper on "Independent Journalism."

(For Dr. Potter's paper, see page 433.)

*The President.*—We have heard Dr. Potter's paper, which I am sure we all appreciate. Before asking for Dr. Jack's paper it would seem best to place Dr. Potter's views before the meeting for discussion.

*Dr. J. B. Rich.*—I think the position taken by Dr. Potter is one that ought to be evident to every one, that before we can have an independent journal, entirely devoted to the interests of our profession, it will need to be separated from the control of manufacturers. That sentiment in the paper is one that everybody ought to respond to. It seems to me absolutely impossible to have an independent journal so long as it is connected with a manufacturing house, and we cannot have a journal that will be thoroughly creditable to the profession until all such interests are eliminated from it.

*Dr. H. C. Meriam.*—It is evident that we are not to be much longer content with a condition of journalism that results in the isolation of dentistry from the other specialties of medicine, and that a journal devoted to its interests should bring to it everything from every source in modern progress in any way related to it. Some years ago a friend wrote me for material I had gathered in working for a society in Massachusetts, and I sent him all I had. He read his paper at one of the large meetings in New York, but in the report of that society's proceedings in a trade journal, that paper was reduced to a single paragraph. Now what could have been the influence of such a proceeding, not merely upon the gentleman who prepared the paper, but on the society that submitted to the editing, and what is the influence on the profession of continued submission to such journalism? How long is this to continue? How can it be helped? Certainly not by agreements in counting-rooms. The way is the way in harmony with our American free institutions.

How can a practitioner hobbled by contract with dealers aid us? He is not a free man; when he comes to our meetings or takes part in the discussions relating to professional life, we find that he is hobbled, that in our counsels he is another man's man. Thus we have the second effect of these journals.

The American Academy of Science is aided by the national government, and in its turn is expected to give aid to the government when information on scientific subjects is needed. Some years ago, I am informed, an inventor of world-wide fame was proposed for membership; but it was pointed out that he was in the pay of large business interests, and that he could not be expected to speak freely on subjects which might injure him or the parties to whom he was under contract financially. Nor could he be expected to give information when he was under contract to sell all he might discover to certain companies.

*Dr. Jacob L. Williams.*—While Dr. Potter was reading his address, I was reminded of a little experience I had some time ago at a meeting, at which I made some remarks quite condemnatory of the practice so general among the so-called parlor dentists, of sacrificing the teeth for the sake of crowning, bridging, etc., which remarks were forcibly seconded by a distinguished Western member. A reporter of a trade journal was present, who was very careful to pick out interesting points to be printed in that journal; but when the journal came out there were none of the condemnatory remarks in the report in regard to that practice. I wanted to trace the thing, and, having some fancy for investigation generally, I made inquiries to find out what proportion of the trade in dental supplies artificial work bore to that relating more particularly to operative work. I was informed, after the matter had been canvassed a little, and I think reliably, that a little less than three-quarters of the trade consisted in what contributed to artificial work. That explained clearly the reason why these remarks regarding the destruction of natural teeth were not considered conducive to the prosperity of the trade. So it is not ourselves only who are interested as a profession, but it is also the public for whom we labor. Our object is to help the public welfare in the best possible way, and it strikes the public through us; if these warnings of injuries are suppressed in such a journal, that journal is not strictly a professional journal.

*The President.*—Will Dr. Ottolengui favor us with his views on this important question?

*Dr. R. Ottolengui.*—Now that I am invited to speak, I must base my remarks upon my practical experience, and not as it seems to me the essayist and those who have come into the discussion have done, from what may be called a high, professional, theoretical, millennial stand-point. In the first place, what constitutes the profession of dentistry? Is it the members of the dental societies, or is it the members only of those dental societies who live up to a high code of ethics? There are about twenty-five thousand dentists in this country; there are, I think, less than twenty-five hundred who are members of dental societies, and I believe there are less than a thousand who have reached the standard of the Harvard Odontological Society, where each man would be either willing or capable of furnishing a paper once a year.

Now, let us suppose that there be a truly professional journal, and that it caters to this elect section of the profession, how much useful work will it do in the world? What proportion of its work would reach the patients through the hands of those who read this magazine? I think a very small percentage. It seems to me that the purpose of the dental journal has not been fully set forth; let us consider for a moment the other ninety per cent. of dentists who are not endeavoring to elevate the profession, or to raise the standard of ethics, but rather to get enough food with which to feed their children. Many men hurry away to a college, very frequently ill fitted even for matriculation, but they get in as best they can, and they get out with as little study as they are compelled to devote to their science; then they are passed by a State board of examiners, and, I may say, "turned loose" upon the community. It is an interesting fact that whereas not more than ten per cent. of the profession generally are members of dental societies, and that only ten per cent. of that number take an active part in the work and management of the societies, that one-half of all the dentists in this country take at least one dental journal. Why? Because all the young men, when they begin practice, discover how much time they have wasted by not studying while they were at college, and it becomes essential to them to find some means of learning more about the practical work of their profession. Will they find it in that highly scientific, millennial journal which has been described, or in that magazine which is managed with sufficient capital so that if some intricate piece of mechanism is to be described, it can be illustrated so that he can comprehend it? Do the members of the Institute think they can to-day start a wave over this country which will support a dental magazine and reach the standard which has been set by some of the so-called trade journals, when it is a certain fact that it will be reached only by a loss of from ten thousand to eighteen thousand dollars per annum? I do not think that our ambitious professional spirit will carry us so deeply into our pockets. And that is the reason why the trade journal has succeeded rather than the other. What has done the work for us, if not the trade journal? Why should we be forever bantering the trade houses that have given us the finest scientific literature in the world? It is not only in dentistry that scientific magazines are difficult to support. I belong to another body of scientists in this city, the entomological; they manage a magazine at an expense to the members of the society, who share the deficit annually; it does not depend upon any trade house, and therefore the cost falls on the society.

Is it true, this constantly recurring statement, that the trade journal sifts everything that comes to its pages in the interests of the owner of the magazine? Long before I was connected with a magazine, I was intimately acquainted with the working of the *Dental Cosmos.* I never found that the editor of that magazine was hampered in any way by the trade house which paid him his salary. If we look around among the editors of this country, who are they? Are they tradesmen, or are they men whom the dental world honors? We have in Buffalo a man who is at the head of a dental college running the magazine of a trade house. In Chicago another man who is respected by the profession manages a magazine owned by a trade house; and our most important magazine is managed by an editor who is the dean of one of the largest university schools in the country. Now, is it fair to these men to suppose that they are controlled by the paltry salaries which they receive, and that they will weed out that which is against the interests of the trade house, even though it be in the interests of the profession? I do not believe that; I have never had a line eliminated from anything that I have written, and it has been not at all infrequent that I have recommended things that were in exact opposition to the interests of the house which published my papers.

Now, the matter of illustrations is not as small as may be imagined. Probably all will admit that the highest scientific class of literature that comes into our magazines is such work as Williams, Andrews, Black, and other histologists have given us in the past, and it would be absolutely unintelligible without illustrations. What would we know of the work these men have done if it had been buried in a little professional magazine whose capital was so small that what these men saw under the microscope could only be given to us by words. I am reminded of the old adage that we should not throw away the stick which has supported us until we are certain that we can get better crutches.

*The President.*—Having heard from both sides in reference to Dr. Potter's paper, we will now give our attention to Dr. Louis Jack, of Philadelphia, who will address us upon the subject, "What should be the Character and Policy of Dental Journalism?"

(For Dr. Jack's paper, see p. 426.)

*The President.*—We have heard Dr. Jack's paper, which is on somewhat different lines from Dr. Potter's, and I hope it will be discussed as it deserves to be.

*Dr. Wilbur F. Litch.*—I have been much impressed by the papers which Dr. Potter and Dr. Jack have read and by the arguments that have been brought forward as to the desirableness of having at least one dental journal in America strictly professional in character, published by men who love truth for the truth's sake, and dentistry for dentistry's own sake,—a journal unbiassed by partisanship and untrammelled by trade interests.

This, as Dr. Ottolengui says, may be a millennial conception, but there must be a beginning to all things, and we have a good beginning in the INTERNATIONAL DENTAL JOURNAL. It is edited with great ability and with absolute dignity of tone, and is in many respects, and to the full extent of its all too scanty pecuniary resources, a model journal. I do not doubt that if it could receive from the dental profession at large that measure of support to which it is entitled it would easily equal, if not surpass, any other dental journal now published.

I say this in no spirit of detraction as to the merits of dental journals published by business houses primarily for business purposes. One of the oldest, perhaps the oldest, of these, the *Dental Cosmos*, has, as we all know, done and is doing magnificent work for dental literature; in the number and value of its illustrations, in the amount of compensation which it pays to its contributors for original work, no strictly independent or professional journal can hope to rival it until at least a far more liberal amount of money is appropriated for that purpose than at present appears possible.

The publication of valuable papers in a journal having a very limited circulation is, as Dr. Potter has very well shown, attended with a good deal of disadvantage, not only to the individual who is seeking a reputation, but to the profession, if they are to be benefited. Almost any man would hesitate, unless he were of a very self-sacrificing character, to spend a great deal of time in original work and have it buried in some obscure journal where it would be seen by a very few people. The matter of illustration is not of minor importance, as Dr. Ottolengui has shown. Not only in the domain of histology, but in the domain of practical operative procedure, illustration is indispensable to clearness of description. Words at best are a lame mode of communicating thought, and when we want to get a tangible thing clearly in the mind of the average man we can best do it by drawing a picture for him.

If, then, we are to have a journal that is not only representative of the profession, but which will benefit the profession, there must be devised a system by which adequate pecuniary resources may be placed at its command. We have an example of what a professional journal may be made by systematic effort in the *Journal of the American Medical Association.* Every member of that association who has paid his annual dues, five dollars, is entitled to receive a copy of this journal free. It is published weekly, and, in addition to the papers read at the annual meetings of the association, contains a large amount of other interesting matter. Some such method as this will be necessary if we are to have a professional journal adequately supported. With the merging of the American and Southern into the National Association the membership should be very largely increased, and if, under the auspices of that Association, a journal were established on the lines of the *Journal of the American Medical Association*, it would give us, I think, what we need.

As to trade journals, I am fully persuaded with Dr. Ottolengui that the publishers very rarely, if ever, interfere with the work of the editorial department, and can hardly think it possible that a contribution would be rejected or changed simply because it happened to recommend a rival process or the product of a rival house. Where larger matters are concerned, however, there seems to be reason to believe that in some cases there has been interference in a direction antagonistic to professional interests.

*Dr. Rich.*—I have very little to say concerning the subject of journalism. My belief is that until the members of the dental profession make up their minds to put their hands in their pockets and pay for a good journal they will never have it. The first effort to establish a journal in this country was the *American Journal of Dental Science*, and I assisted in making up the first subscription list. We had about enough subscribed to publish that journal, without paying the editor anything for his labor, but more than half of those who subscribed did not pay, so that we were not able to get the journal out the second year. I apprehend that that will be the way with any dental journal without proper backing, unless the dentists become more liberal in their efforts to promote it than experience has shown them to have been. There are very few dentists but would say that we ought to have a journal of our own and be free from trade-journalism, but there are many difficulties in the way. The proprietor of a trade journal told me that he had paid high prices for articles from the pen of a member of our profession who would not otherwise have been able to give the necessary time to make the investigations and make a record of the results. The articles referred to are admitted by all to be of the greatest value, and the methods of trade-journalism have in this way contributed greatly to the advancement of the profession.

I am sorry to have to differ with my friend Dr. Jack upon the subject of dental patents, but I do think that it is a very short-sighted policy to deprecate the taking out of patents. I want to know how many of those very valuable aids to our profession would have been on the market to-day if the dentist had not been paid for the labor of originating and perfecting them. It has been announced as reprehensible for a dentist to take out a patent. Now, we would not have had an improved dental chair nor a dental engine, nor hundreds of other things, if the people who had invented them had not been encouraged to take out patents to protect their rights. There is one strong argument in this matter which perhaps those present do not remember. There was a gentleman living in San Francisco who invented a remarkable set of clamps, forty or fifty in number, which he brought to this city and patented. I saw them, and I can say that they were the most valuable set of clamps I have ever seen,—so valuable that when I found they could not be manufactured I offered him one hundred dollars for his set, which he refused. This man applied to Mr. S. S. White to sell the patent, and Mr. White was disposed to give him a very liberal price for it, until, submitting the matter to his attorney, it was found that the patent was not perfect, because there had been a previous application with which this interfered. In the mean time Mr. White found that the whole set of forty could be manufactured for about two dollars, and could be supplied to dentists at a large profit, including a royalty, for about ten dollars, which would have been very cheap. But Mr. White would have nothing to do with them, as a perfect patent could not be granted, and the consequence is that the profession is deprived of the use of these valuable clamps. There are many things which are useful to the profession that would never have been heard of if the inventor could not have patented them, and I think, therefore, that dentists ought not to set themselves against patents. Members of the medical profession do not take out patents, but they write books about their methods and operations and share in the profits of the copyright.

If I spend months in perfecting an invention that is useful to the whole profession, what meanness it would be on the part of those who may use it to say, " We are much obliged to you," and proceed to use it without paying for it. We ought to do all we can to encourage the patenting of articles that will facilitate our operations, and we should be willing to pay the inventor for the use of them.

*Dr. Meriam.*—It seems almost unkind after this paper on " Journalism" to ask our eloquent friend what became of the clamps that were so good and that were shown to the publisher of a journal that could describe them. Is it customary for professional journals to consider ownership or title as a prerequisite before they mention new discoveries in their journals?

There was the description in the Patent Office, and a journal that was devoted to the interests of the professions could publish a copy of the patent as showing advance in dental appliances, and could inform us when the patent would expire and when we could have them made. To illustrate a new set of clamps would certainly not cost more than to prepare the illustrations for papers on other subjects. What kept back the clamps?

*Dr. Rich.*—This man, who had spent years in perfecting these clamps, refused to have them manufactured unless he could be compensated.

*Dr. Benjamin Lord.*—It is well known that I have endeavored to do what I could towards establishing dental journalism upon an independent basis.

My feeling is that we should regard the matter as one of principle and just pride,—that we should not allow ourselves to be dependent upon the journals whose paramount interest is trade for the publication of our professional contributions and the proceedings of our societies. It would seem that we ought to have more sense of independence and professional dignity than to allow such a state of things to exist.

With these views and feelings, however, we need not have any jealousies or any want of respect for the so-called trade journals. They have done good work in editing and publishing important matter, and should have due credit for it; but we are well able to do our own work, and should we not have sufficient pride and self-respect to do it?

It is certainly most gratifying to hear the INTERNATIONAL DENTAL JOURNAL so highly spoken of, for it is felt by many to be one of our most useful journals. All this, as can readily be understood, has not come about without a great deal of well-directed and earnest effort, most of the work having been done by a few, of whom Dr. Louis Jack has stood in the very front and borne much the heaviest part of the care and burden. Dr. J. A. Woodward has rendered him most valuable assistance. All know of the marked ability and fidelity of the editor, Dr. James Truman, and with what hearty interest and faithfulness he has performed his part, and he well deserves our thanks and our high regard.

What the INTERNATIONAL DENTAL JOURNAL now needs is an earnest and increased support on the part of the profession, and the question may very properly be raised, should it not have this support? Should not the profession take the greatest pride in a publication that they may legitimately and properly call their own?

So much good and successful work has been done up to the present that dentists should surely feel under obligations to render the necessary support with a hearty good-will and with the view of not only increasing the number of subscribers, but of aiding in every possible way to make the INTERNATIONAL DENTAL JOURNAL the very best medium of professional and useful information.

*Dr. Ottolengui.*—I should like to ask Dr. Meriam a question. I think he was the presiding officer of one of our largest meetings, held in Boston, in which several States united. At that meeting a very ingenious oven for porcelain work was presented to the profession. I would like to ask Dr. Meriam what ever became of that oven. Personally I have never been able to get one like it.

*Dr. Meriam.*—I suppose that as the description of that oven was never given in any professional journal or book, that it is lost to the sight of the dental profession.

*Dr. Ottolengui.*—Anything that Dr. Meriam sends us for publication will be reproduced with pleasure and free of cost.

*Dr. J. Morgan Howe.*—I am pleased that the statements and arguments are not all on one side, and I am glad that such able representatives of thought, somewhat opposed to what has been presented, are here this afternoon. I think, if I may be allowed to refer to what Dr. Rich said about journals, that he is mistaken in his anticipations as to what will be accomplished. I think that he is rather too pessimistic in supposing that there never will be a very successful professional journal.

*Dr. Rich.*—I beg to be not misquoted. I said there never would be an able professional journal until the dentists put their hands in their pockets and paid for it.

*Dr. Howe.*—I stand corrected; but, in reply, I claim that there *is now* a very able professional journal; the dentists have put their hands in their pockets, and have devoted money and labor to its establishment and maintenance up to the present time. Of course, the amount of support that it has received has been totally disproportionate to these efforts; what they wish is that the amount of support it shall receive shall be more in consonance with its merit. In connection with what has been said in regard to enlarging the circulation and usefulness of a professional journal, it seems a remarkable thing for an argument concerning the support of a journal, that is published by and for a body of men claiming to be professional, to be based on its merits relative to those that are confessedly published for a divided interest, when it is within the power of the dentists to make it superior to anything there is published if they take hold of it. They stand off, each one considering whether he will give it his support,—withholding because it has not had the support they admit it ought to have from others; meanwhile, they give their contributions and other support to its competitors. If even the class of dentists who have been spoken of as numbering something like a thousand, those whose ethical principles are on the highest plane, if even so small a number as that were to give loyal support to professional journalism, without any wavering, there would be no longer a question as to superiority, not only in quality, but in circulation and strength; it would be accomplished at once.

*Dr. W. H. Potter.*—I am glad to see that the discussion is coming down to personal considerations. The last two speakers have indicated just where the weakness exists; it exists in this room, in New York, in Boston, Philadelphia, and Washington. If we wish to establish and maintain a professional journal, we can do it if any considerable number of men in these large cities band themselves together for this purpose. The trouble is that we have divided interests as well as the trade journals. We wish to throw a part of our influence with the trade journals and a small remnant of it with the professional journals, and the latter do not have the support they ought to have.

I do not understand the argument that was made to the effect that the professional journal was not suited to that portion of the profession who may not be as well educated or informed as those whom we commonly meet. That seems to me inconceivable. In our professional journal we do not publish things that are entirely apart from the comprehension of those least educated; there is no reason why it should not be perfectly equipped with all kinds of professional information. It should minister to the needs of the humblest member of the profession.

*Dr. Ottolengui.*—I would like to speak directly to the matter just brought up. When I made the assertion that a strictly professional journal run on millennial lines would not have a wide influence, I meant this, that the field of what might be called mechanical dentistry is very large, more than one-half of the whole percentage of sales in a dental goods business coming from this source, we are informed. That being true, it indicates that a tremendous proportion of practical dentistry is done along prosthetic lines. But if we look over the list of scientists and then examine the articles that they have written in the last ten years we will find how very meagre is the proportion of literature dealing with prosthetics. I know that it takes very many letters addressed to men who are devoting themselves to prosthetic lines to obtain one paper for publication. Another difficulty will be this: Among scientists there is a great deal of what we will call self-respect; a scientist always thinks that he is before everything else a scientist, and he is very jealous of everything he writes; he does not like to have even an adjective changed. Now, it is a peculiar fact that a large number of our scientists have begun the study of science without the fundamental knowledge of English, and it is a hard and onerous duty that the editor has to perform before he can make their contributions palatable. And it is difficult for the editor to forget that a personal friend writes these things. Another trouble in getting support to a professional journal will arise from the fact that the scientists could well afford the price of a subscription to the journal, but they feel that they have attained an eminence in the profession which entitles them to be on the "free list." I met a very prominent dentist recently who made an allusion to something in my own magazine, saying, "That reminds me, why don't you send me your journal?" "Don't you get it?" I asked. He said, "No." "Have you subscribed for it?" He answered, "No; all the other editors send me their magazines." I replied, "The other editors may own their magazines; I cannot send you mine because I do not own it, and, moreover, so far as I am concerned, it goes to nobody free except to students in dental colleges who are trying to work their way up."

I fully agree that it is practicable for us to have a dental journal, and, in spite of the fact that I am championing the other side, nothing would please me more than to see our profession elevated to a plane where it would be possible to maintain its own journal. I understand that the National Association has recently appointed a committee to formulate a scheme for a magazine of its own. The thing to do is not to listen to all the arguments in favor of it, but to just go ahead and see if our thousand professional scientists will agree to pay ten dollars a year for the privilege of receiving *gratis* a magazine which is devoted exclusively to their interests. That would give the editorial department a certain working capital of ten thousand dollars to provide only one thousand copies of the magazine. Regular subscribers would furnish the balance of money needed. If the gentlemen will admit that I am one of the thousand I am willing to pay my ten dollars.

*Dr. S. E. Davenport.*—I desire to be placed on record, and if I say but little it will be because it is difficult for me to give expression to my thoughts on this great subject. The references which have been made to the establishment of professional journals in the past have been most interesting, but those attempts were made at the time when our profession was struggling in its infancy, when every man's hand was raised against his fellow so far as the dissemination of knowledge was concerned, and my wonder is that a professional journal could thrive as well as it did in those days. The dental specialty of medicine has made much progress during the past twenty to forty years, but in this matter of journalism it seems to me not to have kept pace with its advance in most other directions. The trade houses, which were so helpful to us and which had much to do with our progress when we were clinging to any little idea we had in place of giving it to our neighbors, were at that time really more progressive than the members of the profession, and we owe them a great deal for what they did. It seems to me now, however, that since the establishment of the dental trade combination the supply houses have confessed that trade interests are to be first with them, and we ought not, therefore, to allow them to dictate the policy of our journals.

*Dr. Meriam.*—In addition to all other matters of a journal it should be a record of the progress of our specialty. A journal should be so conducted and so in touch not only with the improvements in our own specialty, but, where it touches the other activities of life, that it should be a record of them; it should contain not only the useful matters of the day, but should be valuable as history of our progress. We present papers at our meetings; they are valuable; they show our scientific progress; but when any question comes up relating to the history of dentistry we cannot turn to our journals. If any matter of a patent comes up, we have to go to the Patent Office. In this fight against the Rubber Company, have any of the journals come to the help of the dentist? Have we had any record from the dental journals of various things that were done in the past that have helped us to fight these suits?

Dr. Jack has done well in calling the Combination into court again. Some years ago an explanation was made regarding it, "that any reputable dealer could become a member," but it never was stated how a man became a reputable dealer. In fact, a person who wished to start could not get stock, as the Combination dealers refused to sell to them. Capable assistants or managers wishing to set up for themselves are thus kept from doing so, and though known, perhaps, by all to be honest and upright in all the relations of life, become immediately disreputable if they wish to start as dealers in our supplies.

So, in the answer to Dr. Jack, perhaps the Combination will state just how those who wish to start in dental-supply business can become reputable dealers.

*Dr. G. A. Mills.*—I think that Dr. Davenport has stated the point. The dental profession owes it to itself to sustain a journal of its own. I think that would mark the greatest progress. It is true that we have depended upon the trade journals too long. I cannot pay sufficient respect to the house of S. S. White for the aid which it has furnished, but there has come a time when we owe it to our self-respect to show to the world at large what we can do as a profession in regard to independent journalism. I think we could do ourselves great credit by setting an example of independence. I want to pay a tribute of respect to the INTERNATIONAL DENTAL JOURNAL. I think it a model journal as far as it has gone, and I do not know a man who has ever been connected with a dental journal more able as an editor than Professor Truman. I do think the time has come when we should take a decided stand in regard to this matter of an independent journal.

*Dr. B. Holly Smith.*—I wish I dared trust myself to discuss this question in the dispassionate and logical manner of Dr. Davenport. Though I feel that it is unsafe to attempt that, it would be hardly fair to myself not to add my tribute of praise and commendation to our own journal, the International Dental Journal, and I feel that I should like to be my very best self if I proposed to antagonize the advocates of the trade journals, especially in the presence of so able a champion as my friend Dr. Ottolengui. I think that the consensus of opinion in this audience does not require that I should take this position of antagonism. I simply say that Dr. Mills has voiced my sentiments in this, that there must and will come a time very soon when literary work shall be done by members of the profession, free and untrammelled by any association or any obligation to any business enterprise or establishment. I feel that that is the prevailing sentiment in this audience, and I congratulate you, Mr. President, and the members of this society, that here in New York that thought is foremost.

*The President.*—I have a letter from Dr. Beers, of Montreal, who expresses himself with the utmost enthusiasm over the idea of an independent dental journal which shall fully and freely express all that dentists have to offer, and shall unite the Canadian brethren with those of the United States, with whom at present they are most deeply sympathizing on national lines.

EVENING SESSION.

*The President.*—Gentlemen, we were this evening to consider the subject of "Secrets and Patents." We will first listen to a paper by Dr. Smith, of Baltimore.

*Dr. B. Holly Smith.*—It is probably due to this society that I should say a few words in explanation of the title of this paper, which is put down on the programme as "Professional Atmosphere and Morals." The title of the paper came into my mind without knowledge at the time that the title had been used before, but with a very distinct recollection of impressions that had been made upon my mind by a paper written several years ago by Dr. Meriam. In that paper, I think, the pace was fairly set for this discussion, and therefore I think I do Dr. Meriam no injustice if I try to follow in a feeble and ineffectual way the outline which he has indicated.

(For Dr. Smith's paper, see page 439.)

*The President.*—Our numbers have been augmented considerably since we went to dinner, and so, possibly, a number of those present are not aware that we have become entangled in the question of ethics. Dr. B. Holly Smith has given us his views, and, instead of adopting the plan of this afternoon, I shall ask Dr. J. Morgan Howe to follow immediately with his paper.

(For Dr. Howe's paper, see page 413.)

*The President.*—We have listened with much attention to the reading of Dr. Howe's paper, which is one of a series of several papers from the dental side of the house. Two or three gentlemen from the mother profession have done us the honor to be present to participate in this discussion, and I take the liberty of asking Dr. D. B. St. John Roosa to open the discussion.

*Dr. Roosa.*—Mr. President and gentlemen, I say in the beginning, with no mock modesty, that I am here in the capacity of a learner, in the sense that I have never known until to-night, when I have heard these very interesting and able papers, the difficulties that surround the professional performance of the duties that belong to your specialty. I lament very much, as a member of the medical profession, that there has been any apparent or real separation of the child from the mother. I still think—although that is not a question under discussion—that we specialists—dentists, oculists, aurists, surgeons, and physicians—ought to go together on the same road up to a certain point and then branch off and become what circumstances and the providence of God make us, and advance in the special path which we have chosen. But, still, that is a question, as I have intimated, which will be ultimately settled, and I have no doubt in the right way, and we will not discuss it fully now.

I have to express a personal regret that when for seven years I was a member of the council of the University in this city I labored in vain to attach to the medical department a dental department, and I think I am violating no confidence when I say that the chief difficulty was in the constant reiteration of the medical profession—whether true or not, certainly not wholly true—that dentists did not live professional lives, and that therefore dentists must not be classed with oculists and aurists as members of the medical profession. We have advanced so far in the State of New York in the profession to which you gentlemen belong, the general profession of healing, that we have abolished a written code of ethics, and although there are a few recalcitrants who think—honestly, no doubt—that there can be no ethical propriety unless there is a book of etiquette and a book of laws, yet a vast majority of the profession in this State have upheld the medical standard higher than ever before in the history of the profession, without any written code, and they have during that time made a law which has put the State of New York, as far as admission to the medical profession is concerned, on a higher plane than any other State in the Union except Pennsylvania, which has imitated us successfully. We broke away from the written code of ethics and from the old notion that if a man believes that with a thousand-millionth of a grain of belladonna he could cure scarlatina, he does not know the anatomy and physiology of the human body. We will not quarrel with him because he believes in infinitesimal doses; in that respect we have made ourselves freer. We, without the written code, consult with whomsoever we please so long as the State of New York says that that person is a practitioner of medicine. That may not be new to you, but it is perhaps necessary for me to state it in going on to the brief discussion of the other points. Although we now have freedom in consultations and we do not subscribe to the written code of ethics of the American Medical Association in this State, we are even more ethical and truly professional than ever before.

In this matter of patents and secrets and what pertains to professional manners, the State of New York stands as high as any profession on the face of this earth, wheresoever it may be, in Continental or British Europe or in any State of this country, and far ahead of one of the most cultivated and distinguished States, Massachusetts, where quackery reigns and predominates without legal hinderance, while in New York such a thing as legalized quackery does not exist.

In this paper of Dr. Howe's, let me illustrate, if I may, one of his positions by a medical allusion. Helmholtz invented the ophthalmoscope in 1851. Now, suppose Helmholtz had patented the ophthalmoscope, just imagine the condition of things, the loss to the world. That instrument which he invented is one by which you may see the retina and retinal vessels under favorable conditions, but it was a very imperfect instrument; still it would have paralyzed all attempts to make it a perfect instrument if in the beginning it had been patented. This was a great invention by one of the greatest scientists that ever lived. Had it been patented, we should have settled down and been twenty-five to fifty years behind what we are now, because we have now gone absolutely as far as human skill can go in the perfection of the instrument, without a single patent or any attempt to restrain the ingenuity or liberality of scientific medical observers, and the profession has certainly acquired, in the eyes of the public and the world, a reputation that it could not possibly have had if it had gotten out patents for its discoveries. Mechanics may secure patents, but no professional men. Now, that kind of thing might be continued indefinitely, and there is an illustration between the copyright and the patent. Your books are all copyrighted, but they do not restrict anybody. Suppose Downders, who brought out glasses for the relief of astigmatism, had copyrighted his book and received the money derived from copyrighting it, not to the detriment of any human being, but suppose he had patented,—and I judge he would not have had much difficulty in the United States Patent Office in securing a patent for the cylindrical glasses and the spherical glasses for the relief of astigmatism and hyperopia,—suppose that had been done, what a condition the profession would have been in so far as ophthalmoscopy is concerned! A blight would have fallen upon that part of our science which now, thanks to the unfettered labors of so many liberal-minded and scientific men, has such a healthy and luxurious growth.

Now, Mr. President and gentlemen, it seems to be a question that cannot be debated except on one side, that we as professional men must give the world the benefit of our ideas as soon as we believe they are of any use, so that we may help our fellow-men. We may put them into a book and receive a copyright for the book, we do not prevent the dissemination and full use of it; but if we patent our work, we put a gag at once upon any attempt to open the mouth of the profession on this subject. Suppose there had been a patent upon the proper method or a method for the extraction of cataract, what a position would the hospitals, the dispensaries, and the private practitioners of our profession be in to-day. Suppose there were a restriction on the operation for mastoid disease, when we have the advantage of the experience of a great many men, freely given, spread out on every medical page, suppose there had been any restriction, how much less of a surgeon would every one of us be. And thus you might go on indefinitely. The triumphs of your profession are simply enormous. Coming to the end of the last decade of a tolerably long life, as professional life goes, with a certain capacity absolutely unharmed through the care and skill of a member of your profession, I have a personal appreciation of the triumphs of that profession, which I do not think any man can succeed in over-estimating. I know what dentistry is, and I know that it is perfectly fitting that it should have a most exalted position among the professions, and I think, Mr. President, that if you refuse to tolerate anything which we, in our profession conceive to detract from our high character before the public, from our usefulness to them, you will so much the sooner get that complete appreciation on every hand which you deserve and which has sometimes been denied you.

I am aware that I am not sufficiently acquainted with the difficulties in the dental profession, with its history, to properly and adequately discuss this question; but I certainly can express my complete and entire sympathy with what has been said by Drs. Smith and Howe. I may also express the hope that the day is not very far off when we shall be side by side in the general profession exactly as I sit down now with my fellow general practitioners, although a specialist, and receive the same treatment from them that they expect accorded to them,—in a position of perfect equality. That is what we should have, perfect equality for the dental profession with every other department of medicine and surgery. You may assume you have it, but I think not, as yet; I think you will admit that it is not fully accorded. I think the general impression is that you are to go a step or two further; that you are to completely emancipate yourself from the swaddling clothes of your infancy, when it was thought necessary to protect your great discoveries and your great advances. Here, in this country, the cradle of scientific dentistry; here, in this country, from whence go the dental surgeons that win the respect of the medical profession abroad in the highest degree, and where they are received upon perfect equality, we ought to make the foundations broad and sure, and comprehend in that great foundation a building which shall embrace us all.

I thank you, gentlemen, very much for your very patient hearing.

*The President.*—We are very grateful to Dr. Roosa for his part in the discussion of this subject. I think, however, that he forgot to state one important fact, that both Helmholtz and Downders during the course of their lives realized a degree of respect and consideration on the part of their brethren and the public at large that amply compensated them for their liberality towards their profession.

*Dr. Roosa.*—I may add that I do not think that either of them would have exchanged the position that he held in our profession for the fortune of a Vanderbilt, had it been possible to obtain it through patents, which they never secured and never tried to get.

*Dr. Robert H. M. Dawbarn.*—I had hardly realized until I heard these papers by Drs. Smith and Howe that there could be any discussion on such a matter in this year of grace. I did not have the pleasure of being present this afternoon and do not know what was said at that time, but it seems to me that it would be a good deal like opposing some of the propositions of the decalogue to oppose such ideas; and he must be truly archaic in his mental attitude who would wish to take the profession back to the time when secret medicines and secret methods and medical devices patented for personal profit were approved of at all.

In discussing only to-day, at the library of the Academy of Medicine, with one of the most distinguished members of the Academy the paper which I knew Dr. Howe was to read to-night, he used an illustration which I cannot refrain from giving. He told a little tale of a very modern little girl who heard her mother tell her for the first time the story of Pandora and the box of ills, and the gentleman said that the illustration reminded him somewhat of what the dental portion of our profession are now discussing. The mother was carefully going over the story, endeavoring to work up the climax, and the small daughter listened intently until the mother had reached the point when Pandora could not resist the temptation to open the box for the ills to fly out, and then the little girl broke in,—"I know what flew out! GERMS!" She was unquestionably up to date. And, gentlemen, probably in the whole box there are no germs more subtile and injurious to the profession in innumerable ways than the advertising methods and the secret devices which the more advanced members of it have to face and to fight. It is not alone in this country, it is not by any means because we are a new country, that this battle has to be fought. I often think we are farther advanced in these regards as a profession than is the profession abroad, certainly in continental Europe. I could give a dozen instances, where two will suffice, to illustrate this point. It is only a few years ago that one of the most famous pathologists in Germany, Dr. Weigert, advised, as a means of attacking tubercle bacilli, a scheme of killing them in the lungs by inhaling intensely hot air. It is well known that tubercle bacilli are easily affected adversely by a moderate increase of temperature above that of the body, and he had an arrangement for rendering the air intensely hot and permitting its inhalation from behind an asbestos shield. He patented it in Germany and brought it over here with the intention of making a lot of money out of it by having the profession take it up. He was accompanied by strong recommendations from prominent men in Germany. After it had been used a little while here it proved a complete fizzle, and Dr. Jacobi remarked with sarcasm that it was evident that tubercle bacilli were more easily killed in Germany than here. Now, the point is, in regard to that apparatus, that not for a moment would such an attempt have been tolerated here. The inventor who thus violated his oath, taken at graduation, to give freely for the good of humanity any discovery of his, would have to a certainty been censured by a committee on ethics. Take France for a second instance. It is not over two years since Professor Fort, of Paris, came over here and demonstrated at the New York Academy of Medicine his method of cutting through a urethral stricture by an electrical device, thereby effecting a very rapid cure, and a French gentleman, a very prominent physician here in town, a most reputable member of the profession, told me that Professor Fort was very much bent on patenting that device and getting up a company in this country which was to make an enormous profit upon the cost of the instrument. And this gentleman told him that if he did so all the other French physicians in New York would denounce him and withdraw their support. Professor Fort is a member of the École de Médecine in Paris; so that it is plain that we in this country are not alone in our struggle for the right in these matters. A distinction must be drawn, however, between those doctors who patent for personal profit and those who do so to protect the profession and the public. I could name several instances where an ingenious surgical device has been patented by its inventor, who thereupon gave the patent freely to the profession. In this way he prevented some unscrupulous dealer from patenting and then overcharging for his (the doctor's) instrument.

By way of showing you that there is a feeling among us that physicians should always keep before their eyes the fact that they would be disapproved by the better members of their profession if they were to yield to inducements for personal profit in these matters; and to allude for a moment to the other branch of this topic, —that regarding drugs,—let me say, and those gentlemen who are members of the County Medical Society will bear me out, that the County Medical Society invitations are always printed with a standing quotation from the by-laws strongly disapproving of any member attaching his name to a recommendation of any nostrum or proprietary medicine, or anything of that kind. Let us avoid even the *appearance* of evil. Experience has shown that if the members were allowed to forget that regulation, some of them would perhaps have occasion to be reprimanded, but by continually keeping the law and the gospel before them such a necessity may be avoided, and it seems to me that it would not be out of the way for the dental societies to take a similar step.

So fully has the matter been discussed both by the distinguished gentlemen who have just spoken and by the writers of the two papers, and so little do I wish to take the time of those who may follow,—especially as I do not feel moved by the spirit to say anything new, but merely to uphold the hands of those who are making this good fight and who have the hearty sympathy and approval of all other branches of the medical profession,—that I will conclude simply by remarking that when the chapter of true medical progress is written up I have no doubt that the page devoted to your work will show a distinct ethical advance achieved; and every man who has taken this attitude will feel proud that he has done so, since thereby he has demonstrated that he has had the true interests of our profession at heart.

*Dr. Rich.*—I was surprised at the contents of the two papers read this evening by gentlemen who occupy a distinguished position in our profession. Both have been benefited to a great extent by the improvements that have been made because of the patent laws in the apparatus we use, but they advise the profession to set their faces against the taking out of patents on the ground of professional etiquette. I would like to know who, among all in this room, would like to have the many things they use which have been patented taken away from them and be left to their own resources. No other one thing has assisted in the advancement of this profession so much as have the patent laws. Our method of operating has been entirely changed, our facilities for performing skilful operations have been increased a thousand-fold, and this is due in a great measure to the operation of the patent laws in stimulating improvement in the machinery and apparatus that we use. What would the men of to-day do without the dental engine? and that is not at all in the condition that it was when it was first presented to the profession; the patent laws have not prevented hundreds of improvements upon it. Look at the hand piece; the patent on it has not prevented improvements. Who will pretend to say that any person in this room would have devoted the time and energy that were required in order to arrive at the perfection which the hand piece of the engine has reached to day and give it to the profession without compensation?

See what an advantage the patent laws have been to our country. Look at the mowing machine; this was first presented in the crudest form, but the fact of its being patented did not prevent improvements upon it. That is a mistaken idea. Other illustrations are the reaping machine, the ditching machine, and the sewing machine. Look at the improvements in our dental chairs. We had at first to operate with chairs that had no movement at all; but gradually one patent upon another has been added, the obtaining of the first patent not preventing the after-improvements, and now we have a chair in which we can make the patient comfortable in any position we desire. Why should we ignore the right of the inventor to secure his invention and the profits of it?

Regarding the cases that Dr. Roosa has spoken of, I think I have already proved by my illustrations that if there had been a patent on that apparatus for examining the eye the patent would not have prevented improvements. I do not want to be understood as advocating secret nostrums or processes or the patenting of methods; I simply speak of the patenting of instruments and apparatus.

*Dr. James McManus.*—The subject of patents does not interest me, perhaps, as much as it ought. The Bible says, "God hath made man upright, but they have sought out many inventions."

For years I have paid money to fight invalid patents and to protect men who would not give one cent to protect themselves. I paid to fight the Vulcanite Company, and during that time I never made or had any one make for me one piece of vulcanite. I belong to the Dental Protective Association, and perhaps in the future I may continue to pay to fight patents. For years I have earnestly tried in an humble way to fight the claim to any honor of one patentee, and I had, fortunately, the majority of the dentists in the country with me.

I was amused last year at the efforts made by a dental journal in asking the dental profession to pay honor at this late day to the pioneer patentee of a secret anæsthetic nostrum.

The older gentlemen present will recall to memory "Letheon." I merely allude to this to show that patent annoyances started years ago, and they bid fair to continue. While the workers in this direction have my sympathy, I hope they will temper their enthusiasm, so that under no circumstances, as has been said by one editor, will they be liable to render the profession an object of ridicule. The business side of dentistry has been rapidly pushed to the front of late years, until now the special and attractive feature of many dental meetings is the exhibit. We may need all the new devices and appliances and the notorious secret nostrums that agents supply in such alluring packages, and that a trade journal had offered as an inducement to increase its subscription-list, but one can hardly keep back the thought that a powerful search-light may yet be needed if we wish to pierce the foggy atmosphere that seems to be gradually settling down and enveloping dentists and dentistry. Much that is good can be said of dentistry as a trade or business, but the atmosphere of professed professional dental journalism may well give one the chills and fever; and it is unfortunate that this condition—for it is a "condition and not a theory"—is one that we cannot very well shake off. We have to thank God for a few journals that sustain a professional reputation, and they ought to have a more generous support. We have others that claim to be professional, but their pages often give evidence that their editors have no conception of what the words prudence or professional mean. It was highly professional for the editor of a trade journal recently to put forth the strong statement in reference to State officials that, "So long as they aim to fulfil their duty, they are entitled to respect, and any disrespect to them is antagonism to law itself;" but in a later issue of his journal he fell from grace when he permitted to appear letters that contained slanderous and libellous charges against five honest men, who had aimed to fulfil their duty as dentists and State officials.

Journalistic carelessness, and consequent violation of the code of ethics, was illustrated again in the recent allusion by the editor of a trade journal to the action taken by the Connecticut State Dental Association regarding advertising anæsthetic nostrums, not, as erroneously stated by the editor, *local ancæsthetics.* The other imputations cast upon the honesty of the members of the Association might or might not be entitled to some consideration on the part of his readers if the editor had given publicity to the name of his informant.

Editors have rights, privileges, duties, and very grave responsibilities, and if they are members of the national and other associations they are bound to maintain the code of ethics. The members of these associations have also rights, which they should assert in season and out of season, and one of those rights, unfortunately for the professional tone of dentistry, has too often been unasserted. It is our duty to give support and praise when they are deserved, and also our duty and our right to criticise and condemn the editors of dental journals, and trade journals professing to be professional, whenever they allow to appear in the journals they edit any editorial, any communication, correspondence, or questionable advertisements that will tend to lower the professional tone of dentistry.

They should be looked up to as the guardians of the honor of the profession, and they, of all men, should "avoid everything calculated to discredit or bring dishonor on the profession." If they fail in this respect, no special privileges should be granted to them, and they should not be permitted to take up the time of associations by reading their papers or to take part in any of the discussions. It is time that the code should be respected and enforced fearlessly, and that all dentists who claim the privileges of membership in professional associations, whether they are editors, professors, commissioners, or examiners, or men of wealth and prominence in the community, should be brought up with a quick turn if they are found transgressing the code. If this is not done, the term professional is a misnomer and associations are a failure. The code of ethics embraces and includes all that is professional and moral. The man who lives up to its requirements must and will be a gentleman and a Christian in the fullest and broadest acceptation of both those terms. To my mind, one of the causes of the low moral and professional tone so noticeable to-day is that all of us have shirked a duty. We have suspended the code like the sword of Damocles only over the heads of many hard-working men striving to earn enough to support a family, while editors, professors, and men supposed to have wealth have been allowed to transgress not only the spirit but the letter of the code without reprimand or censure. Dentistry to-day is suffering, as the country is suffering, from a combination of malign influences: trade methods and the pushing to the front of low-priced materials, the "jingo," "get-there-at-any-price" element, "cheap-john" establishments, and the illustrated yellow-toned character that has been gradually creeping into journalism. All must have noticed what our lamented good old friend Dr. Atkinson used to call the "retrograde metamorphosis" that has been going on for some time, and, while it is to be regretted, we still may foster a hope that the influence of The New York Institute of Stomatology and kindred associations throughout the country, working along the same line, may before the century runs its course clear the professional atmosphere.

*Dr. Potter.*—I want to say only one word, and that is in regard to anæsthesia. It seems remote from this subject, but I am one of those who believe that Dr. Morton had an important part in the introduction of anæsthesia, and I think that one reason why he failed to get the credit that was due him for his share in the work was because he undertook to patent his process. We know very well that it was not long before there were rival claimants, and they seemed to come up when financial considerations were brought strongly before their minds. I believe if Dr. Morton had given out freely the result of his work he would have received the full credit for it.

*Dr. C. T. Stockwell.*—There are two points of Dr. Howe's paper which seem to me absolutely fundamental and all inclusive, regarding the whole matter under discussion. These points are embraced in the following quotations from the synopsis of his paper, which was kindly submitted to me in advance:

1. "The ethical tone of dentists needs elevating."

2. "Professional character depends on moral relations of practitioners to one another and to the public, not on kind of work done."

In regard to the first quotation, it may be said that this is a statement of universal application. It is a need that is felt in all professions, in all strata of life, throughout society as a whole; and it is a fact that should afford us encouragement rather than the reverse. It means that the *ethical sense* in humanity is surely growing. It means that the ethical standards of the past do not satisfy us to-day. It means that the world is moving towards higher ideals of human relationships. So I say that the very fact that we are so conscious that the ethical tone of dentists needs, and so sorely needs, elevating is one of the encouraging signs of the times. It surely means an upward-tending professional life.

It is doubtless true that the question of ethics, so far as the average mind goes, both in and out of the professions, is a question surrounded with much of vagueness in its apprehension. It does not lie in the common mind with any clearness of definition. Because of this fact Dr. Howe has done us a service of marked value, it seems to me, in formulating a perception of the real professional character in a manner so clear-sighted and comprehensive,—viz., "Professional character depends on moral relations of practitioners to one another and the public, not on kind of work done."

Let us examine this statement with some care. Allow me also to express the hope that it may mark a new epoch in the progress of our profession.

For half a century past, more or less, the chief field of interest and work has been along scientific lines. The "stars" in our profession have been its scientific workers. Consequently we very naturally have come to regard such men as *types* of professional character. We have failed to perceive that a deeper law underlies this question. Even now, I apprehend, it may be questioned by some whether the emphasis should be placed on moral relations or on scientific efficiency, on skill in operating, or on ethical observances with one another and the public. The fact is, however, that true moral relations imply and include efficiency in knowledge and quality of work done. The ideal dentist or professional man is one who is both scientific and ethical. He may, however, be thoroughly scientific and at the same time very unethical,—in other words, in no sense professional.

Let us try to express in as few words as possible the real meaning of these two terms, the scientific and the ethical. Or, rather, let us bear in mind the distinction implied when each term is used. With what realm of thought or fact does each, in an exact way, deal?

Science, as we all know, is correlated with what can be dealt with by the methods of observation and experimentation. Science deals with the *facts* of man and his environment. Ethics, on the other hand, deals with purely ideal conceptions, which can neither be seen nor handled, nor experimented with, and are true to the mind alone.

If our end and purpose in life be to secure the happiness and prolongation of human existence, we shall find on examination that these ends of ours have their genesis not in science, but in ethics. Science may and does inform us as to what means we must use to secure our ends; but as to what our ends *should be*, as to the supreme rules of action, we must go deeper than science. Conscience is ever thundering in our ears, "Do right." But it never tells us what *is* right or what is bad. These are relative terms. The *ought to be* in all the relations of life has never been tested, has never been verified by actual experience, and so lies beyond the plane of the scientific method. Perfection in any realm of life has never been actualized in human experience, and so has not been verified in fact, and is not verifiable in the strict scientific sense. Nevertheless we *believe* in high moral ideas, not because we have seen them fully and completely embodied in any given individual, but because of their own intrinsic attractiveness and authority. There is *something* in man that responds to these ideals —call it what you will—and evolves the ethical sense. Hence arises ethical progress.

As the ethical sense develops and becomes refined, the ethical standards of yesterday are found wanting when measured by the ethical requirements of to-day. Thus the facts, the actualities of existence ever lag behind the ethical ideals that shape themselves in man's apprehension.

And so it appears that beyond the realm of *what is*, the scientific, ethics opens the realms of *what ought to be.* Science deals with men as they *are*, while ethics deals with men as they *ought to be.*

Viewed in this light we at once perceive that a vast distance stretches away between one point and the other,—between the general fact of experience on one side, and the moral imperative on the other.

We also see that a high ethical ideal is the nerve of progress. History shows not only that every great and masterful mind has its genesis in some deep conviction, but also that such minds express that greatness by striking out for the thing in which they so strongly believe. Such a man is dominated by his ideal in all his actions. It gives point and purpose to life. It nerves those who, to quote a reformer's words, "rise from the lap of artificial life, fling away its softness, and startle you with the sight of *a man*."

The supreme question then for each is, What is the ideal of life, and how shall it be pursued? Here we come to the foundation of all ethics, and as are a man's ethics, so is his life, for no life can rise above its ethical convictions.

The oft-repeated question as to whether life is worth living must find its answer in the related question, What is life for? If money, power, social position, our own pleasure and happiness merely, are what we believe in and live for, life may very likely not prove to be worth living. But if our conviction is that life has a high ideal, and that our highest aim is to realize it, then life takes on an incalculable dignity and worth.

Those of us who are familiar with the literature of the scientific world to-day cannot have failed to notice that it is conceded on all sides that the ethical life, in its completeness and perfection, is the logical, the inevitable goal of that process of the world's on-going termed evolution. It is towards this end that all the forces of the universe are seen to be steadily and surely tending. This is the *ideal* that is the inherent and most real of any grade of "realism."

A recognition of this fact explains why it is that the study of ethics occupies the attention of the world's best minds to-day as never before. Ethical societies, ethical literature, ethical professorships abound increasingly. Old systems are being discarded and recast continually. The very best brain and heart of the world are engaged as never before in an effort to readjust and elevate the ethical standards, and to apply them to all the various phases of our complex life. The study of political economy and of economics is to-day considered an essential in even business and commercial life. These studies involve the deeper problems and principles of ethics. Men are *forced*, willing or otherwise, by the exigencies of business life to consider the questions of ethics. And the fact is being increasingly borne in upon the minds of all thinking men that there is no health to the individual apart from the health and well-being of all. The human family are to-day brought so near together, and interests are so interwoven in all directions, that if one suffers all must suffer. Not only social and economic interests, but moral interest and well-being are dependent upon standards that are common and in a large sense universal. Herbert Spencer formulates this conception in the following pregnant statement: "No one can be perfectly free till all are free. No one can be perfectly moral till all are moral. No one can be perfectly happy till all are happy."

But what application, you may say, has this line of thought to our profession, its duties, and privileges?

I answer as follows: The principles and laws that I have thus briefly considered are fundamental laws and principles, and underlie our profession in the same way that they underlie life in any and all forms. Any standard or code of ethics must rest, therefore, upon that enlightened conception of what *ought* to constitute the relations of man with man,—not that which is merely "practical," but that which is ideal.

There is, in fact, but one ideal for all of us, either as members of a profession or as members of society in general, *his highest self*, —highest in moral purpose and aim, and highest in the power to do, to execute, to *actualize* these moral ends.

This is the law written in each and every soul. It is absolute and universal. We do not and cannot choose it. It chooses us, and makes its logical pursuit the highest possible attainment. To set up any idol, of whatever nature, in place of this ideal, is to violate the highest law of our being. It is founded in the nature of things; and nature ever stands ready to vindicate her own laws.

This is nature's method. What has been our course? In all our attempts at legislation we have, thus far, entirely lost sight of the true basis of the professional character. We have attempted to base it on science or scientific efficiency. Such candidates for practice as have successfully passed our scientific examining boards we have vouched for as safe, reliable, true, professional men. They stand before the public equally sanctioned and endorsed by the high authority of the State. There is no practical discrimination whatever on the only real grounds of professional standards,—viz., moral relationships and ethical aims. It may have been, and probably was, legal for one of our State examining boards to license a convict who was, at the time the license was issued, serving his sentence in the State's prison. But what a burlesque of ethical standards!

From the stand-point of professional character, our State legislation is thus far worse than utter failure. Much, it is freely admitted, has been gained on the purely scientific side. But this gain, desirable as it is in itself, is more than offset in the practical outcome of these laws by a grossly lowered state of the professional status. Seven-tenths, shall I say, of those who pass our State boards are, in practice, actuated simply and solely by motives no higher than that of personal commercial gain. The State, while vouching for their scientific efficiency, does not provide, by any effectual means whatever, for their professional conduct. It does not guarantee the public against non-professional treatment at the hands of these men. They may possess the requisite knowledge to serve the public properly. But when we scan the newspapers, the street signs, etc., we know that the only object is money, and money alone, utterly regardless of the best welfare of those who, unfortunately, fall into their hands.

True, sadly true, it is to-day that the one great need of our profession (if indeed we may call it a profession) is the elevation of its ethical tone. Let us, if need be, rest upon our oars for awhile, along scientific lines, and concentrate all our energies upon those moral and ethical aspects which alone give us any claim to a place among the honorable professions.

*Dr. Meriam.*—We seem to overlook one point. That is, what we secure by *not* patenting: the advantage of association, the unconstrained professional life and intercourse. Our friends here in active medical practice will bear me out in saying that if operations were patented consultation would be impossible. It is a great pleasure that this question has broadened since we took it up some years since. Liberal professions are not liberal by vote, but because freedom is essential to their members in ministering to the needs of mankind. Even our patent advocates would not call or employ a physician who was not free to treat them, or one who was obliged to obtain the consent of another physician before he could prescribe a remedy or use an instrument, but would insist upon a man free to use anything for their good.

Then the freedom in imparting information is important, in open meetings and papers, and the social professional intercourse. Contrast this with the barred doors, curtained windows, and stuffed key-holes of the patent worker. "No Admittance" is the sign displayed at the door of the inventor's workshop. It is not seen on the doors nor does it show itself in the lives of men who make up such a society as this. I am glad that Dr. Howe has brought out the subject of copyright, for though it seemed sufficient years since to point out that copyright had never been used in medicine to limit its freedom, yet it was a point behind which many took shelter who did not see that the answer was all around them in schools and libraries.

Some years ago I was asked to report what I had done in investigating the subject of gutta-percha. I went to the Public Library in Boston, stated my object, and was taken into one of the alcoves, given a table, and a boy brought me the journals and books containing matter relating to the subject. This is going on in all the libraries in the country. In fact, a few earn a livelihood looking up subjects, and the points thus gathered enter into new books, papers, and addresses.

Contrast this with the continual "law suits," "interference," "infringements," etc., over machines that are patented at every joint or part and the secrecy in which the inventor works. I doubt if the inventor spends more on his models than you, gentlemen, spend in getting up papers, specimens, and all that makes a professional life possible.

There is the rule that the conduct of members should be such that if universally imitated would result in the highest good of all; contrast this with the practice of a body which should sell to parties not of it each discovery that it might make. What would be the result if a boy sold part of his inheritance when he required anything to supply his needs? He would soon toil as a workman in fields that his father loved or sleep as a hireling 'neath the roof to which he had been heir.

*Dr. Shumway.*—I do not think I ought to take up the time at this late hour; certainly it was wholly unexpected, and it is rather a strange place to put me, for I am a patentee. About seventeen years ago I took out a patent, but if one takes out a patent that is not good for anything he does the profession little harm, and as about nine-tenths of the patents never amount to anything we are not in such great danger as we imagine. This is a very fruitful theme for discussion, and I must say that I sympathize with what

Dr. Rich has said. We may be in danger of shutting ourselves up and preventing progress, but it will not do to stop where we are. While we have a great many inventions, and some valuable ones, if we should stop now I am inclined to think that dentistry would be rather a failure. I was in Providence a week or ten days ago, in the office of one of the dental examiners of the State of Rhode Island, a very fine man indeed, and he deplored the condition of dentistry, saying that "Rhode Island was overrun with 'Cheap Johns.'" "What," he said, "is to be done? The more law we have the worse off we seem to be; we do not know what the outcome is to be. We ought to have some sort of law obliging a man to put his name over his door instead of 'New York Dental Parlor' or 'Boston Dental Parlor,' etc., where no responsibility is fixed at all upon the individual." He further said that "it is lowering the standard of dentistry and doing us an immense deal of harm." If we stop now we are in danger of going down-hill instead of going up to the high plane where we desire to be. As I said, it has been a source of financial loss to me to be a patentee. It was a most unfortunate thing for me that I conceived the idea of doing things a little differently from everybody else. If one stands alone his patients may look upon him with distrust. I have had them say to me, "You do not do this as Dr. —— does. Why, he filled my teeth with a heavy lead mallet. I do not think it possible that you can do good work with such a slender instrument as that of ivory. Will you please put in something temporary as a stopping." Alas, for my patent! If a man takes out a patent it does not follow that he will become rich out of the profession. There is no money in a patent unless it happens to be a good one, and very few of us ever get a good one. Now, I do not wish to be understood as saying that a thing may not be good in itself. I am speaking of patents from the money stand-point. What I have patented I believe to be of very great value. In fact, it changes cohesive gold from being "the most dangerous material for filling teeth" to the best material for tooth-preservation. Nothing can be more valuable in my judgment from the professional view. A patent may bring large returns, but it must be of a kind to meet present needs, without too radical a change in methods. This is what I mean by saying it was unfortunate for me to be an inventor and patentee. I would express exactly what Dr. Rich says, that I do not think any professional man has a right to patent a nostrum or any process of curing disease or putting upon the market any secret remedy.

The President requested the Secretary to read the following letter from President Eliot, of Harvard University:

"HARVARD UNIVERSITY, CAMBRIDGE, MASS., March 31, 1898.

"MY DEAR SIR,—I have always held that the dental profession should be assimilated to the medical. Now, it is a main characteristic of the medical profession that it gives away without price every discovery which it makes, and that it admits no such thing as a secret remedy or practice. It is therefore my opinion that a reputable dentist should never take a patent on any instrument or material used in dental practice; and, moreover, that a reputable dentist should not trade in dental supplies.

"My opinions on this subject are doubtless influenced by my long conversance with University practices and ideals. The University discovers and invents, but invariably gives away every discovery and invention. In every medical and dental school the teachers are constantly training up young men to supplant themselves. They are bringing forward their own successors, and, as they hope, making them their own superiors in knowledge and skill. This is the truly liberal practice; and if the dental profession expects to win the title of a liberal profession, its reputable members must conform to these professional standards.

"Very truly yours,

"CHARLES W. ELIOT.

"E. A. BOGUE, M.D."

The following is an extract from a letter received from Dr. W. W. H. Thackston, of Farmville, Virginia:

"I am with you and The New York Institute of Stomatology, heart and soul, in all your plans and efforts to redeem all American dentistry from disgrace and shame of 'patent rights,' 'secret methods,' and 'quack nostrums.'

"For more than half a century I have been fighting for a position alongside and on the plane occupied by the 'learned and liberal professions' and against all men and measures that dishonor dentistry as a legitimate department of science, and wherever I find men working for that end and object I am with them."

*The President.*—It seems, gentlemen, that the subjects for consideration this evening have been, as Dr. Howe expressed it not very long ago, floating in the air. At the end of the constitution of the National Dental Association, organized last August, occurs a resolution reading as follows: "*Resolved*, That the National Dental Association condemn the use of secret preparations known as 'local anæsthetics,' as well as other secret preparations."

In the January *Forum* is an article, I think by a lawyer, deprecating the taking out of patents, and the grounds of his argument are very much those of Dr. Shumway. He was answered by Elisha Foote, who was formerly commissioner of patents, but the answer is not as forcible as the original article, and it narrows itself down to very much the same lines of expediency. One of our eminent surgeons said to me the other day, "It is a good thing to talk about ethics; a good thing to advocate high ethics. You have my hearty sympathy, and you shall have my hearty support; but you might just as well expect to convert the world to Christianity in short order as to expect to convert it to an altruistic doctrine like that. Go ahead; every step you take is to your advantage and the advantage of your patients."

The Institute directors the other evening appointed a committee to draft a resolution. That committee has done its work, and I would ask Dr. Davenport to present the resolution that it may be considered.

Dr. Davenport read the resolution referred to, which is as follows:

WHEREAS, It is evident to us that the conditions required for professional progress are now the same as they were when our honored predecessors established a periodical, journal, a society, and a college as means of interchanging ideas, disseminating knowledge, abrogating secrecy, and raising the required standard of education; and, whereas, our periodical literature cannot subserve its highest function if owned by manufacturing and supply houses, notwithstanding their enterprise, because their interests are not always identical with all lines of professional advancement; and, whereas, the common practices of withholding knowledge concerning the formulæ of compounds, and of patenting inventions and discoveries, operate to prevent the general diffusion of information and to deny freedom to put new ideas into tangible form; therefore, it is hereby resolved that we deprecate the use of secret compounds, and would urge dentists everywhere to dispense with their use as quickly as they can find other compounds of known composition to take their place.

And it is also resolved that dentists who manufacture or have an interest in secret compounds, or who patent their inventions or discoveries relating to the treatment of diseased or abnormal conditions of the human body, are retarding the progress of our profession, and it is also resolved that we would respectfully urge upon all College Faculties and the Association of Dental Examiners of the various States and the National Dental Association the need of considering present professional conditions and of adopting such measures as will bring about a change in the views and practices of dentists and students in regard to journalism and to secrets and patents.

We would hereby entreat all dentists to support by all means in their power the journals we have, not owned by supply houses, to the end that they may be made stronger and have the means to do their work of spreading knowledge and building up professional moral character more effectually; and it is hereby resolved that copies of these resolutions, signed by the President and Secretary, be sent to the faculty of each Dental College and to the Association of Dental Faculties and to the Board of Examiners of each State and to the National Dental Association.

The resolution was unanimously adopted.

*Dr. Smith.*—It seems only fair, in taking any position, that the person should have an opportunity to defend such position against those who assail him. If it is your pleasure to close the discussion of the subject, however, I would not wish to interfere with that.

*The President.*—Dr. Smith is correct, and I beg to apologize. He and Dr. Howe should have an opportunity to make reply to those who have spoken against them, and I have to request Dr. Smith to do so.

*Dr. B. Holly Smith.*—I wish to say that at no time have I taken a position which I do not intend to defend, and here my good brother from Washington brings the serious charge that a man of respectable character has placed himself in an absurd situation by advocating the absence of business methods and the doing away with the commercial spirit in professional matters. I feel that I would like to "get back at him."

My impression is that we have been entirely too tolerant in these matters. I will not answer his argument as to the disadvantage which he claims the absence of patented appliances would be to the progress of dentistry, because I do not believe, and I do not think there are three persons in this room who believe as he does, that the absence of patents on such appliances would stop the study and invention of these appliances. But I do want to say that, for myself, I would rather practise dentistry according to what I believe are the holy traditions of my profession, and do my duty according to these traditions, than to use all the patented appliances which have been devised for the use of dentists. I speak for myself in that, but I would much prefer to live up to these sacred traditions, which I believe is my full and absolute privilege, than to place myself under obligations to these commercial schemes and plans. I am reminded of a little incident. Smith said to Brown, "Have you any rats and mice in your house?" "Yes," answered Brown. "Well, what do you do for them?" Probably remembering the check he had to send to the Society of Antiquated Cats, to which his wife belonged, he said, "Do for them! I give them a place to live, and all they want to eat and drink. What do you expect me to do for them?" Now, that is what we do for these patentees. We give them a place at our meetings, receive them on an equality, and what else are we expected to do for them? I am glad to appear before the society to defend purer and holier methods. I recall the Song of Solomon:

> "Awake, oh north wind; and come thou south;
> Blow upon my garden, that the spices thereof may flow out,"

and I think that in this little conference we have had here we may invoke all influences towards holier and better thoughts, and send them forth to the uttermost parts of the professional atmosphere to spread purer and higher ideas.

*Dr. Howe.*—I do not think it is desirable for me to take up much time. I would just remark that the argument in favor of patents completely ignored the points made in opposition. It is not a question of whether or not we are willing to go without new appliances, but whether they should be obtained, with or without detrimental and damaging influences. Some improvements made on patented devices are not proofs that other improvements are not forbidden. It is sometimes advantageous to a patentee to make or permit improvements, but the power to prohibit and to shelve is inherent in patents, and is constantly used.

Adjourned.

S. E. Davenport, D.D.S., M.D.S.,
*Editor The New York Institute of Stomatology.*

